# Design and Characterization of Ocular Inserts Loaded with Dexamethasone for the Treatment of Inflammatory Ophthalmic Disease

**DOI:** 10.3390/pharmaceutics16020294

**Published:** 2024-02-19

**Authors:** Omar Rodrigo Guadarrama-Escobar, Cassandra Araceli Valdés-Alvarez, Karla Stella Constantino-Gonzalez, Pablo Serrano-Castañeda, Ma. Concepción Peña-Juárez, Miriam Isabel Morales-Florido, Mariana Salgado-Machuca, Betsabe Rodríguez-Pérez, Isabel Marlen Rodriguez-Cruz, Dinorah Vargas-Estrada, Crisóforo Mercado-Márquez, Alma Vázquez-Durán, Abraham Méndez-Albores, Ericka Anguíano-Almazán, José Juan Escobar-Chavez

**Affiliations:** 1 Unidad de Investigacion Multidisciplinaria L-12, Facultad de Estudios Superiores Cuautitlán, Universidad Nacional Autónoma de México, Cuautitlán-Teoloyucan, Km 2.5 San Sebastian Xhala, Cuautitlán Izcalli 54714, Mexico; escobaromarrodrigo@gmail.com (O.R.G.-E.); 417092623@cuautitlan.unam.mx (C.A.V.-A.); karlaconstantino@gmail.com (K.S.C.-G.); pabloqfb@hotmail.com (P.S.-C.); maconcepcionpenajuarez@gmail.com (M.C.P.-J.); mflorido.cf@gmail.com (M.I.M.-F.); salgado518.12mariana@gmail.com (M.S.-M.); eri.qa.30@hotmail.com (E.A.-A.); 2 Laboratorio de Farmacia Molecular y Liberación Controlada, Departamento de Sistemas Biológicos, Universidad Autónoma Metropolitana, Xochimilco 04960, Mexico; 3 Unidad de Investigación Multidisciplinaria L-6 (Laboratorio de Servicios de Análisis de Propóleos), Facultad de Estudios Superiores Cuautitlán, Universidad Nacional Autónoma de México, Cuautitlán Izcalli 54714, Mexico; betsarguez79@gmail.com; 4 Unidad de Enseñanza e Investigación, Hospital Regional de Alta Especialidad de Zumpango, Carretera Zumpango-Jilotzingo # 400, Barrio de Santiago, 2a Sección, Zumpango 55600, Mexico; isabelmarlen05@gmail.com; 5 Departamento de Fisiología y Farmacología, Facultad de Medicina Veterinaria, Universidad Nacional Autónoma de México, Ciudad de México 04510, Mexico; dinorah.vestrada@gmail.com; 6 Unidad de Aislamiento y Bioterio, Facultad de Estudios Superiores Cuautitlán, Universidad Nacional Autónoma de México, Cuautitlán Izcalli 54714, Mexico; crisofo@gmail.com; 7 Unidad de Investigación Multidisciplinaria L14 (Ciencia y Tecnología de los Materiales), Facultad de Estudios Superiores Cuautitlán, Universidad Nacional Autónoma de México, Cuautitlán Izcalli 54714, Mexico; almavazquez@comunidad.unam.mx (A.V.-D.); albores@unam.mx (A.M.-A.)

**Keywords:** dexamethasone, ocular inserts, centra composite design, ophthalmic route

## Abstract

The short precorneal residence time of ophthalmic drops is associated with their low absorption; therefore, the development of ocular inserts capable of prolonging and controlling the ophthalmic release of drugs is an interesting option in the design and development of these drugs. A surface response design was developed, specifically the Central Composite Design (CCD), to produce ophthalmic films loaded with Dexamethasone (DEX) by the solvent evaporation method having experimental levels of different concentrations of previously selected polymers (PVP K-30 and Eudragit RS100.). Once optimization of the formulation was obtained, the in vivo test was continued. The optimal formulation obtained a thickness of 0.265 ± 0.095 mm, pH of 7.11 ± 0.04, tensile strength of 15.50 ± 3.94 gF, humidity (%) of 22.54 ± 1.7, mucoadhesion strength of 16.89 ± 3.46 gF, chemical content (%) of 98.19 ± 1.124, release of (%) 13,510.71, and swelling of 0.0403 ± 0.023 g; furthermore, in the in vivo testing the number and residence time of PMN cells were lower compared to the Ophthalmic Drops. The present study confirms the potential use of polymeric systems using PVPK30 and ERS100 as a new strategy of controlled release of ophthalmic drugs by controlling and prolonging the release of DEX at the affected site by decreasing the systemic effects of the drug.

## 1. Introduction

The eye is the structure in charge of concentrating light and focusing it onto photoreceptors, which allows it to convert it into electrical impulses towards the visual cortex where the sensation of vision takes place. The sensation of vision can be divided into the ability to detect light and movement, visual perspective, visual field, depth perception, visual acuity, and colour and shape perception.

It has recently been recognized that the pathology of age-associated degenerative eye disease such as adult macular degeneration, glaucoma, and diabetic retinopathy have strong immunological underpinnings, and systemic inflammatory disease commonly affects the sclera, cornea, retina, and orbit, and can pose a serious threat to sight [1,2].

Despite their side effects and the advent of systemic immunosuppressive and biologics, the use of corticosteroids remains in the management of patients with uveitis. Corticosteroids as a local therapy for uveitis is well stablished, but periocular injections of corticosteroids can also be used to control mild or moderate intraocular inflammation [3].

Traditional ophthalmic administration in aqueous drops is characterized by its low bioavailability in addition to rapid precorneal elimination. To obtain therapeutic concentrations requires frequent instillation of the drug, which leads to low compliance with ophthalmic therapy [4], which is a recurring reason for the poor therapeutic results in eye pathologies. The development of topical bioactive formulations capable of overcoming the low bioavailability of conventional eye drops is critically important for the efficient management of ocular diseases [4,5].

### 1.1. Nasolagrimal Duct System (NDS)

The lacrimal duct system transmits tears from the surface of the eye to the nasal cavity. The NDS consists of a secretory component (Precornean Lacrimal Film (PLF)) and an excretory component. Tears enter the duct system at the lacrimal punctae and conduct through canaliculi within the eyelids. The canaliculi drain into the lacrimal sac. Obstruction of the lacrimal duct system results in epiphora or excessive tearing. This condition is particularly common in children but is also common in adults [6,7] (Nasolagrimal drainage system) [8]. The PLF is formed as follows: a three-layer structure of the lacrimal film such as a mucous layer, a layer of watery tear, and a lipid layer. Preocular lacrimal film provides the smoothest surface refractive optically for the cornea, which is essential for a defined visual image. It is resistant to gravitational forces. It must be stable so that it remains continuous between consecutive blinks and must be able to repair itself. A continuous and normal tear film also plays an important role in protecting and maintaining the well-being of the corneal surface and provides adequate lubrication for the eyelids without the superficial lipid layer [9,10,11].

The NDS consists of the upper and lower puncta, the paired lacrimal canaliculi, the lacrimal sac, and the nasolacrimal duct [6].

The lacrimal sac and the nasolacrimal duct are lined by a double-layered epithelium and are surrounded by a wide-ranging vascular system that is comparable to a cavernous body [6,12].

### 1.2. Eye Immunity

The mucosal immune system defends the eye surface from antigenic attack. This immune function is mediated primarily through secretory IgA (S-IgA) antibodies, which are known to inhibit viral adhesion and internalization and prevent adhesion, colonization, and bacterial activity; they also interfere with parasitic infestation and reduce antigen-related damage to mucosal sites. Therefore, the immune system of the ocular mucosa exists to protect the eye from allergic, inflammatory, and infectious diseases, thus promoting the integrity of the conjunctiva and cornea to preserve visual acuity [13]. Ocular immunity is highly specialized, so there is a regional immune response that obeys eye mechanisms to maintain homeostasis and minimize potential damage by immunogenic inflammation. It has been considered an immunoprivileged region as is defined by [7,14], as follows.

1. Active and passive immunomodulation in the microenvironment.
2. Ability to alter the function of immune system cells.
3. Immunological tolerance for tissue transplants.

### 1.3. Ocular Inflammatory

Eye inflammation is the result of various aggressions including accidental or surgical trauma, exposure to toxic substances, infection agents, non-infectious immune stimulation, and various physical agents, in addition to neoplasia [15,16,17,18]. In any tissue, cell death triggers an inflammatory reaction aimed at removing dead tissue. There are a couple mechanisms by which cell damage promotes inflammation: the release of chemicals (mainly prostaglandins) that operate as direct inflammatory mediators, and the release of inflammatory mediators (primed cells) and activation of slow mediators within the plasma. The inflammatory reaction is a beneficial physiological mechanism limited only to the immediate area of injury, and for this reason, the inflammation should exhibit a significant degree of moderation and specificity so that there is no damage in the surrounding tissues or global effect on the health of the animal [15,17].

### 1.4. Clinical Pharmacology and Therapeutics

In eye therapy, drugs should be sought not only based on their pharmacodynamic or pharmacokinetic properties, but with the best route of administration, which complies with the dosage regimen to maximize the desired effect [16]. Within the ophthalmic structure there are different areas that require medication (Table 1) [19]. Sometimes it is difficult to release drugs because the eye has severe structural protection mechanisms such as blinking, permanent lacrimal secretion, and drainage, which are necessary to preserve vision but quickly remove the drugs that are administered. Factors to consider when attempting ophthalmic administration include [15]:

(1) How to cross the outer layers of the eyeball and blood barriers to reach the inside.
(2) How to locate drug action in eye tissue by minimizing systemic effects.
(3) How to prolong the duration of the drug’s effect so that the frequency of administration is minimized.

### 1.5. Dexamethasone

Dexamethasone (9-fluoro-11β, 17,21-trihydroxy-16a-methylpregne-1,4-diene-3,20-dione) is a synthetic derivative of glucocorticoids and is characterized by its high potency. Among the corticosteroids used in ophthalmic therapy, dexamethasone sodium phosphate (DEX) has stood out for its high potency and effectiveness [20]. The action of DEX is mediated by the binding of the molecules of the drug to glucocorticoid receptors present in various cells [21].

### 1.6. General Characteristics

**Figure 1:**
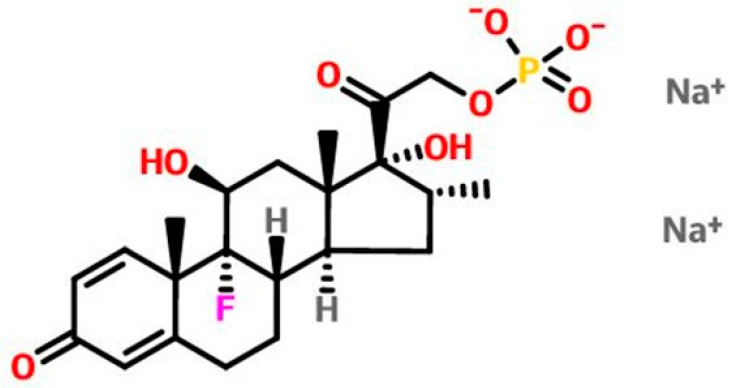
Dexamethasone sodium phosphate chemical structure.

1. Molecular structure: C_22_H_28_FNa_2_O_8_P (Figure 1).
2. Molecular weight: 516.4 g/mol.
3. Physical characteristics: it is a white powder, crystalline, painless, stable in the air; excessively hygroscopic.
4. Solubility: easily soluble in water, slightly soluble in methylene chloride, and poorly soluble in ethanol [22].

## 2. Materials and Methods

### 2.1. Material and Equipment

Beaker with magnetic stirrer (Ultraturrax IKA, Wilmington, NC, USA), analytical balance, drinking trough and cages of acrylic, Mettler Toledo DSC882e, scanning electron microscope (SEM JSM 6010LA, JEOL, Dearborn Road, Peabody, MA, USA), Spectra-Pro membrane dialysis tubing 45–50 KDa (Spectra/Por, Miami, FL, USA), Labwit ZWY-103D shaker-incubator, ultra-centrifuge, HALO DB-20 UV–Vis double-beam spectrophotometer (TechComp, Mexico City, Mexico).

### 2.2. Preformulation Studies and Selection of Polymers for Matrix Formation

Preformulation studies were conducted on the selection of the components to make sure they follow the characteristics of the drug and to test the compatibility between them, based on their physicochemical characteristics that allow the control of the release of DEX at the application site by reducing loss from nasolacrimal drainage, thereby significantly improving bioavailability. Moreover, the selected polymers have proven to be compatible with biological systems and are therefore suitable for use as controlled-release pharmaceutical forms.

The polymers selected in different mixtures are described in Table 2 and were chosen from previous studies that confirm their use by an ophthalmic route.

### 2.3. Development of the Ocular Inserts

Once the polymer mixtures that formed films were selected, the composition of the formulation was determined and working conditions were established to standardize the method of preparation. The ophthalmic inserts were made using the solvent evaporation technique.

### 2.4. Design of Experiments (DOE)

A surface response design was developed, specifically, the Central Composite Design (CCD) to produce the ophthalmic films loaded with DEX by the solvent evaporation method having experimental levels of different concentrations of the previously selected polymers (PVP K-30 and Eudragit RS100) to provide various characteristics to the product.

### 2.5. Physicochemical Characterization of the Ophthalmic Films

#### 2.5.1. Weight Variation (WV) and Thickness (Th)

For the determination of WV and Th, 10 circular films with a diameter of 1cm were randomly selected and individually weighted on an analytical balance and the average and standard deviation were calculated. On the other hand, the thickness of the films was obtained with a digital calibrator measuring different parts (three) of the pharmaceutical form to determine the average and its standard deviation.

#### 2.5.2. pH

The pH measurement was performed with the potentiometer HANNA HI2210, United Kingdom, once the components of the mixture were completely dissolved (10 min) in 3 different points of the mixing vessel (surface, midpoint, and bottom).

#### 2.5.3. Tensile Strength (Ts)

**Figure 2:**
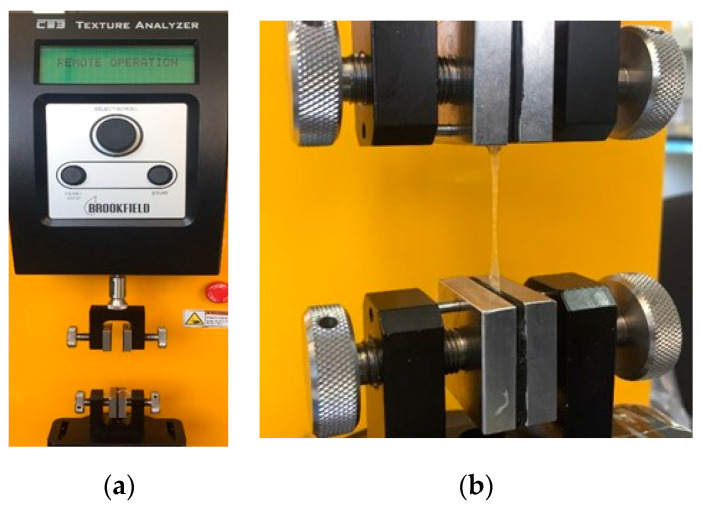
Evaluation of mechanical tests: (**a**) texture analyzer CT3 Brookfield^®^; (**b**) sample placement to measure stress at break using the TA-DGF attachment.

The voltage properties were measured with the Texture Analyzer CT3 Brookfield, USA using general test parameters: an activation load of 6.8 at a speed of 0.5 mm/s. Each formulation was evaluated in triplicate, and samples were cut with the following measurements: 10 mm long × 5mm wide. The samples were held by the TA-DGF attachment positioned according to the equipment requirements (Figure 2).

#### 2.5.4. % Humidity (Hu)

The % humidity was taken as the weight loss at drying, for which a sensitive thermobalance was used to weigh the sample and an infrared lamp to dry. The technique was based on placing a sample portion in the thermobalance, which is initially weighed, exposed to a set temperature depending on the type of sample, and left for a period of time. Three ophthalmic inserts (OI) of each formulation (F1–F11) with a diameter of 1 cm were weighed in a PMC50 USA, redwing thermobalance to determine the % of humidity.

#### 2.5.5. Mucoadhesion Strength and Mucoadhesion Time Ex Vivo (MS and MT)

The mechanical tests performed are important because they allow us to evaluate the physical behavior of the pharmaceutical form, that is, if it has the characteristics necessary to withstand the wear and tear of its daily use. Eyes were obtained from the necropsy laboratory of the Facultad de Estudios Superiores Cuautitlán-UNAM. Once extracted, they were placed in an FSS solution (0.9%) for transport and storage. The test was carried out in the first 24 h from the extraction to preserve the anatomical structure. The test was performed on 2 anatomical structures: the eyeball and conjunctival sac.

##### Mucoadhesion Strength (MS) Ex Vivo

The samples were placed in glass petri boxes and 50 μL of Simulated Lacrimal Fluid (SLF) [23,24] was added to each sample to simulate the actual conditions of the anatomical structure.

Once the sample was placed, the MS was measured using the Brookfield CT3 texture analyzer under the following conditions: target value 50 g, activation load 0 g, and test speed 1 mm/s.

Each formulation was evaluated in triplicate by placing it at the base of a cylinder contacting the structure.

##### Mucoadhesion Time (MT) Ex Vivo

**Figure 3:**
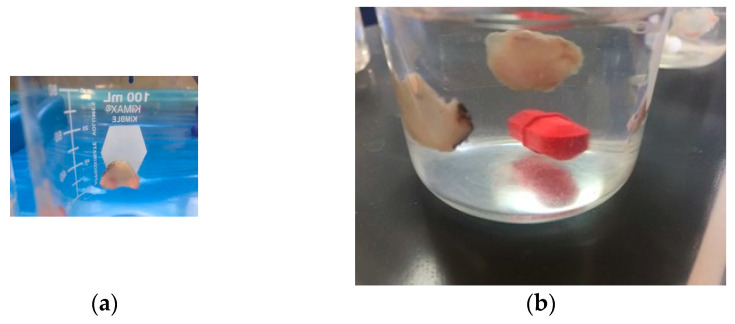
(**a**) Conjunctival sac cuts (1 cm diameter) placed with double-sided tape on the wall of a beaker. (**b**) The formulation is attached to the mucosa and the test was performed at the mucoadhesive test time.

For the determination of the ex vivo mucosal adhesion time, portions of the conjunctival sac (approximately 1 cm in diameter) were placed on the walls of a 100 mL beaker with double-sided tape; in the area of the conjunctival sac were placed the various formulations and later the vessel was filled with 50 mL of SLF (Figure 3) and the test was carried out at 50 rpm and 37 °C to evaluate the time that the mucosal-bound formulation remained [25].

#### 2.5.6. Swelling (Sw)

For the Sw test, three samples of each formulation were cut with a diameter of 1 cm, which were weighed in an analytical balance (W1), then immersed in 5 mL of SLF pH 7.4 0.1, and placed at 37 °C. Each sample was weighed every 10 min as follows: the sample was removed from the DES by removing the excess of it with Whatman paper to be weighed later (W2) [26,27]. The swelling was determined through the weight variation in the samples; once this ceased to vary, it was indicative of the completion of the test, and finally the formula described below was used:

Hi = ((W_2-W_1)/W_1)

#### 2.5.7. Chemical Content (ChC)

To determine the chemical content of the formulations, samples of 1 cm in diameter were cut and dissolved in 1 mL of ethanol, later transferred to a volumetric flask of 25 mL, and the solution was read by UV–Vis spectrophotometry (Cary 100 Varian, Santa Clara, CA, USA) at a wavelength of 243 nm [17].

#### 2.5.8. In Vitro Release

This test allowed us to gain an idea of what the behavior of the drug would be once placed in the placement site. Samples of 1 cm in diameter were cut and placed in 50 mL beakers with 20 mL DES pH 7.4. The conditions of 50 rpm and 37 °C were constant for 5 days of sampling. Then, 1.5 mL samples were taken during the established times. The samples were analyzed at a wavelength of 243 nm in the Cary 100 Varian spectrophotometer in order to obtain the release profiles of the DEX loaded in the OI.

### 2.6. Optimization and Statistical Analysis of Formulation

The data obtained from the mechanical and physicochemical tests were analyzed using the StatGraphics Centurion XV statistical program to obtain formulation optimization.

#### 2.6.1. Optimization of Formulation

Having obtained the optimized formulation, it was characterized according to the tests mentioned below.

#### 2.6.2. Ex Vivo Permeation Test

The optimal formulation was tested using Franz cells. For the studies, eyes obtained from the FES-Cuautitlán Necropsy-MVZ Laboratory at Cuautitlán Izcalli Estado de México, were used as biological material, to learn the following biopharmaceutical parameters: flow, permeability constant (kp), and latency time (LT).

**Figure 4:**
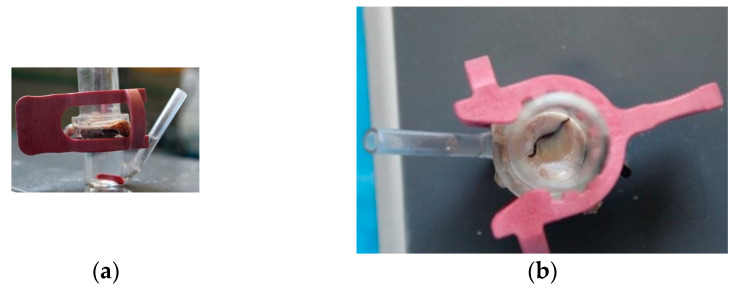
(**a**) Franz cell type used for the study of permeation ex vivo. In the upper part is the donor compartment and in the lower part the receiver. (**b**) Top view of Franz cell where the formulation placed on the biological structure is observed.

In the receiver compartment, 7 mL of SLF pH 7.4 were added and a magnetic bar was introduced. The optimal formulation was placed on the conjunctival sac and both compartments were sealed with constant agitation at a speed of 50 rpm at 37 °C for 48 h (Figure 4). A sample of 1.5 ml was taken from the receiving compartment by replenishing the same volume. The samples were analyzed spectrophotometrically at a wavelength of 243 nm.

### 2.7. Sterility Testing

As culture media, Triptyasein Soy Agar (TSA) and Tioglycolate Broth (TB) were used. For sowing in TSA, a scrape was made with a sterile swab on both sides and the edges of the OI, and for the TB, an OI of 1 cm in diameter was placed in the liquid medium. Sterility kinetics were carried out by subjecting the films to different radiation times emitted by a UV lamp (0, 10, 20, 30, and 60 min) and subsequently the formulations were placed in both culture media to be incubated at 30–35 °C. They were observed for 14 days to determine microbial growth.

### 2.8. In Vivo Test

For in vivo tests, the research protocol was submitted for review and approval by the Internal Committee for the Care and Use of Experimental Animals of the Facultad de Estudios Superiores Cuautitlán (CICUAE-FESC) belonging to the UNAM, with registration key CICUAE-FESC C 19_01.

For this last stage, 20 New Zealand albino rabbits of indistinct sex with a weight of approximately 1.5–2 kg were used, which were clinically inspected by the MVZ who performed a general vision examination to rule out the presence of ulcers, corneal damage, alteration of the lacrimal film, or the indication of the development of an inflammatory process. The 20 animals were transported from the Cuniculture Module (FES-Cuautitlán) to the UIM-FES Cuautitlán isolation and vivarium unit and were given an adaptation period of 2 weeks.

### 2.9. Determination of the Inflammation Produced by Instillation of Arachidonic Acid (AA) and by the Placement of the Ophthalmic Insert (OI)

The first experimental part consisted of the random selection of 4 rabbits for the conduction of a pilot study that allowed for determining the duration of the “Inflammation model in rabbits by Arachidonic Acid induction” [28,29]. At the beginning of the test, a tear fluid (TF) sample was taken to know the basal levels of the animals. To complete this, 100 µL of SSF were instilled in the lower sac of the rabbit’s eye and mixed gently to then recover as much as possible. Subsequently, 50 µL of AA prepared in SSF were instilled in concentrations of 0.05, 0.1, and 0.25% in the right eye and the left eye was used as a control, instilling 50 µL of FSS. After 10 min, the first sample was taken as previously described and the next sampling times were after 1, 2, 4, 6, 10, 12, 15, and 17 h.

To determine the irritation caused by the OI, the OI placebo was also placed in the right eye and the left eye was assigned as a control. The methodology used was the same as for determining the concentration of AA.

For each sample collected, the migration of polymorphonuclear cells (PMN) was evaluated by diluting 10 µL of FL with 10 µL of Turkes liquid to count the number of PMNs in the Neubauer chamber. When the number of PMNs returned to the baseline level, fluid samples were not taken as it was possible to determine the duration time of AA-induced inflammation and inflammation caused by placebo.

### 2.10. Comparison of the Anti-Inflammatory Effect of DEX in Drops (OD) and Inserts (OI)

The rest of the animals were randomly divided into 2 groups (N = 8) with free access to water and food as follows.

- Group (1): Conventional treatment by instilling OD of DEX.
- Group (2): Alternative treatment by administering OI of DEX.

In both groups, a sample was first taken to know the basal levels of each subject and the left eye was selected as a control, instilling 50 µL of FSS, and in the right eye the different pharmaceutical forms were tested as follows.

- Group (1): 50 µL of DEX solution was instilled in drops (1 drop).
- Group (2): The ocular insert was placed in the lower sac of the rabbit’s eye.

After 10 min of treatment, 50 µL of AA was instilled at 0.25%. The evaluation of the model was carried out based on the following tests.

1. Clinical evaluation: inspection for damage, signs of irritation, changes in pupil response, etc.
2. PMN migration: Assessed in the same way as in the pilot group.

## 3. Results

### 3.1. Selection of Polymers

Mixtures of ophthalmic polymers were made in different percentages (Table 3) to determine the ability to form films.

### 3.2. Composition of the Ophthalmic Insert and Standardization of the Preparation Method

From the results obtained in the preformulation studies, the polymers and the rest of the excipients (Table 4) were chosen for the preparation of the ocular inserts.

### 3.3. Design of Experiments

#### Central Composite Design (CCD)

This explains the factors, levels, and responses to be evaluated in the design of experiments that consisted of a star-shaped central compound design (Table 5 and Table 6).

### 3.4. Physicochemical Characterization and In Vitro–Ex Vivo Evaluation

The results obtained after the physico-chemical evaluation (weight, % humidity, pH, resistance to rupture, etc.) and the in vitro and ex vivo tests are shown in the Table 7 and Table 8. These results were analyzed by the statistical program that allowed us to obtain the optimal formulation in terms of the characteristics desired by us.

### 3.5. Design of Experiments

**Figure 5:**
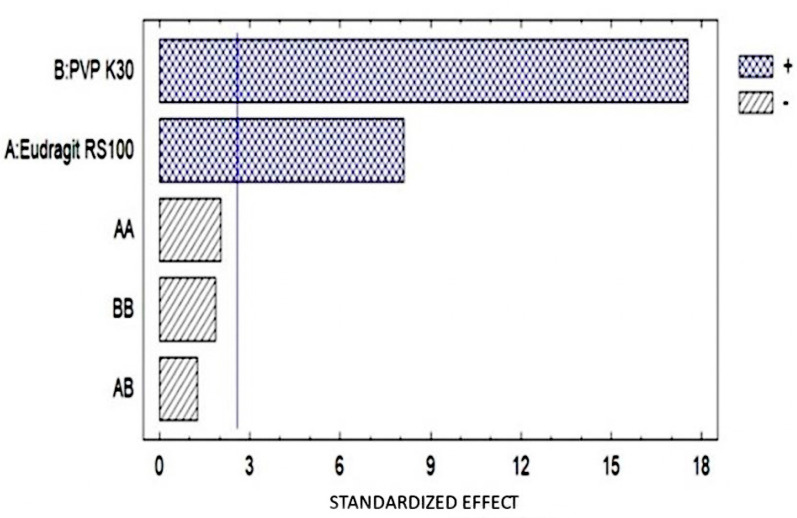
Pareto diagram for mass standardization. The diagram shows each of the estimated effects in descending order of magnitude indicating that for WV, the % of PVP K-30 and ERS100 are statistically significant in a positive manner.

The inserts were cut with a diameter of 1 cm and from each formulation 10 were randomly chosen to determine their average weight (Figure 5).

### 3.6. Thickness Variation (ThV)

**Figure 6:**
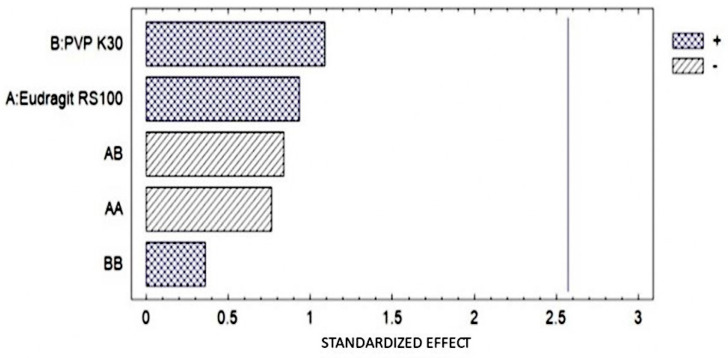
The diagram of ThV shows that all values are below the vertical line, so they are not statistically significant with a 95.0% confidence level.

To determine the ThV, 10 samples of each formulation were measured at different points, obtaining the results in Figure 6.

### 3.7. Formulation pH

**Figure 7:**
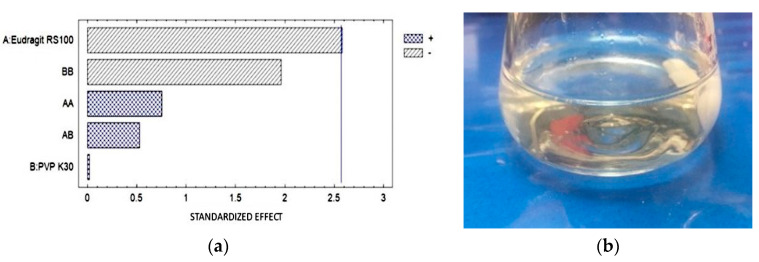
(**a**) The Pareto diagram for the pH determination shows that E RS100 polymer has a significant effect on OI. (**b**) Solution of ophthalmic film.

The pH measurements were made using a potentiometer to establish that this characteristic is within the value reported to be used by the ophthalmic route (Figure 7).

### 3.8. Tensile Strength (TS)

**Figure 8:**
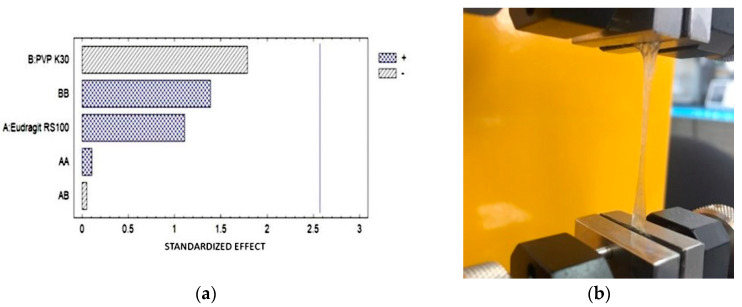
(**a**) Pareto diagram showing that no factor has a significant effect in the formulation. (**b**) Elongation of the ophthalmic film during the test.

The determination was carried out by selecting 3 samples randomly, obtaining the Pareto diagram. Furthermore, it was possible to observe the maximum elongation achieved by the OI (Figure 8).

### 3.9. Humidity (Hu) (%)

**Figure 9:**
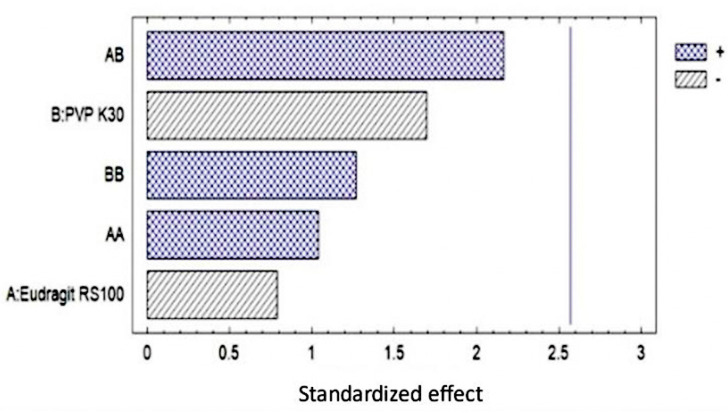
Pareto diagram for humidity results. There is no statistically significant difference.

The humidity of the OI was determined in the thermobalance, obtaining an average result of 27.1% humidity for the formulations. In Figure 9, it can be observed that none of the factors present had statistically significant effects.

### 3.10. Mucoadhesion Strength (MS) Ex Vivo

**Figure 10:**
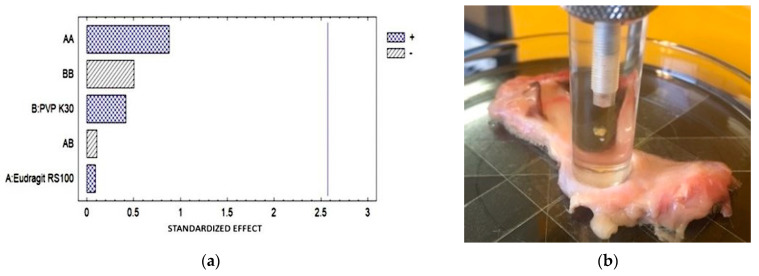
(**a**) Pareto diagram shows the effect of each factor on the measured property, as well as the interactions between them; (**b**) elongation of the ophthalmic film during the test.

The ex vivo MS was performed using eyeballs and dog conjunctival sacs (Table 9) obtained from the Necropsies Laboratory of FES-Cuautitlán UNAM. In the Pareto diagram (Figure 10), it is observed that there was no statistically significant difference; however, the greatest interaction was related to the concentration of Eudragit RS.

### 3.11. Chemical Content (ChC)

**Figure 11:**
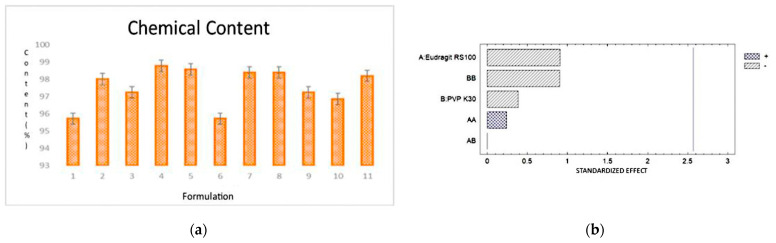
Results of the chemical content. (**a**) Graph showing the % ChC in a range between 95.71 and 98.77%; (**b**) Pareto diagram showing that there is no statistically significant difference.

The tests of chemical content were carried out according to the current Mexican regulatory norm: in this case the AP content of each formulation was compared by UV–Vis spectrophotometry in order to know if they were within the established limits (90–110%). The evaluated formulations (F1–F11) had a chemical content in a range between 95.71–98.77% and the differences between the content of each formulation were not statistically significant (Figure 11).

### 3.12. In Vitro Release %

**Figure 12:**
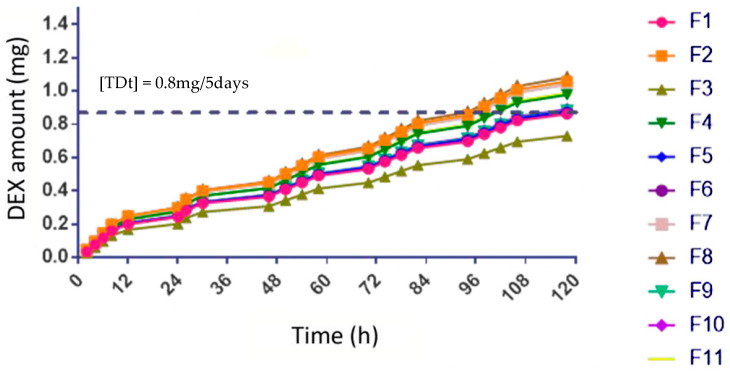
Results of AP release from the OI; the dotted line represents the theoretical therapeutic dose considering ophthalmic administration every 2 h.

The release test was performed by placing the formulation in FSS to quantify the % of drug released from the polymer matrices. The determination was made by UV–Vis spectrophotometry, obtaining a release of 12.5%. (Figure 12).

### 3.13. Swelling (Sw)

**Figure 13:**
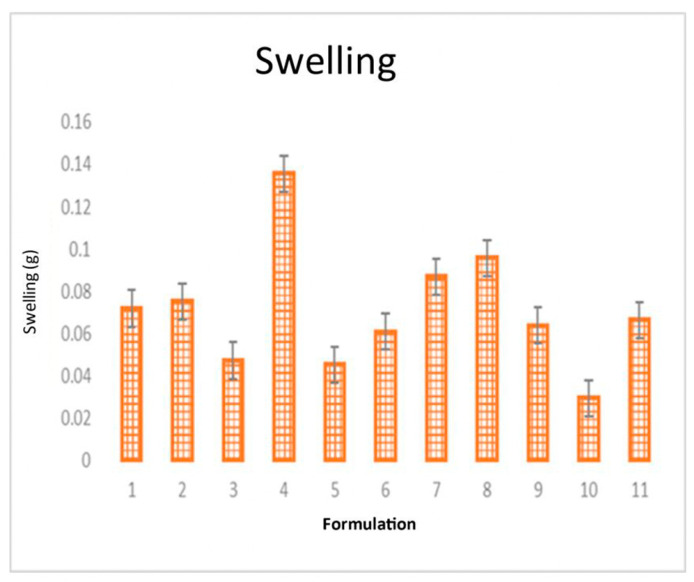
Variance results of % swelling with an STF retention average of 0.02 g.

The Sw is a test that indicates the movement of polymer chains that allow the incorporation of molecules from the surrounding medium, in this case, DES at 37 °C; samples were taken during 10 min intervals to obtain a constant weight. The Sw results showed that the weight increased in the different formulations in a range between 0.02–0.13 g (Figure 13).

### 3.14. Optimization of Formulation

**Figure 14:**
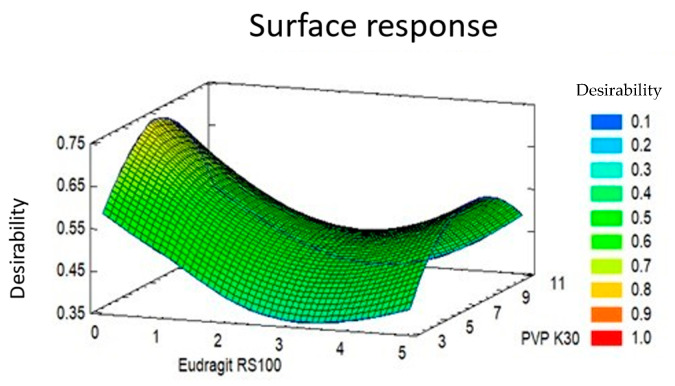
Response surface diagram resulting from the optimization process of OI.

After analyzing the data obtained in the physicochemical characterization, formulation optimization was performed; for this purpose, ThV, MS, DS, and Sw response variables were chosen. In this way, an optimal value of desirability equal to 0.719274 and optimal quantities of each polymer were obtained to obtain formulations with the desired characteristics (Table 10, Figure 14).

Based on the response surface graph obtained after the optimization test (Central Composite Design), the OI was prepared with the standardized method and, therefore, was characterized physiochemically according to the tests mentioned in the methodology, obtaining the results found in Table 11. The results showed that the OI have the physico-chemical characteristics suitable for use in in vivo testing.

### 3.15. Permeation (Ex Vivo)

**Figure 15:**
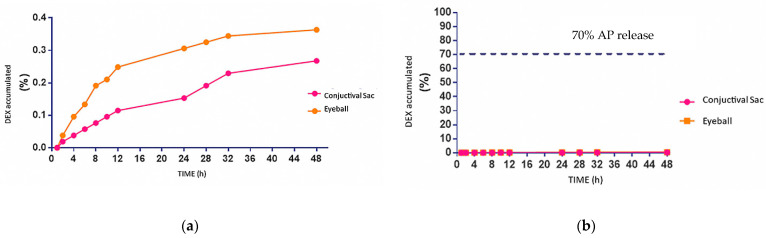
(**a**) % quantified DEX in the receiving compartment of cells, type Franz; (**b**) % of DEX accumulated during ex vivo permeations. It is noted that the amount quantified is not close to 70 percent.

After physiochemically evaluating the optimal formulation, the ex vivo permeation study was carried out using the conjunctival sac and the eyeballs as anatomical structures in order to obtain the amount of accumulated DEX (Table 12) contained in the receptor compartment of the Franz-type cells, determining that for 48 h the accumulated percentage of DEX is 0.26% in the conjunctival sac and 0.36% in the eyeball. Likewise, the purpose of this test was the kinetic evaluation of the uptake of the drug and its diffusion through biological structures; however, due to the low amount of drug released (less than 70%) (Figure 15), it was not possible to obtain the kinetic parameters corresponding to latency time (h), flow (µg/cm^2^/h), and Kp (cm^2^/h).

### 3.16. Sterility

Ensuring sterility is one of the critical parameters for those pharmaceutical forms with ophthalmic route administration. The test was performed by taking a portion of the OI of 1 cm in diameter, exposing them to UV radiation for different lengths of time, then sowing them in culture media (TSA and CB) for 14 days at a temperature of 30–35 °C.

**Figure 16:**
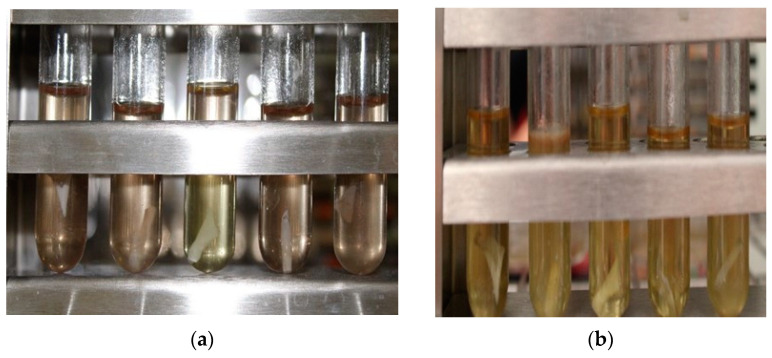
(**a**) OI at the beginning of the test; (**b**) observation of OI after 14 days of incubation at 30–35 °C showing the development of microorganisms at times less than 20 min of UV radiation.

Microbiological growth was measured (Figure 16) so that it could be established that times greater than 20 min of UV radiation inhibit the growth of microorganisms (Table 13).

### 3.17. In Vivo Test

The effectiveness of the pharmaceutical form in an animal model (New Zealand albino rabbits) was evaluated using 20 subjects. It was determined that during all tests the left eye would be used as the control and the right eye for the different treatments.

### 3.18. Determination of AA Concentration, Duration of Inflammatory Model, and Inflammation Caused by OI

**Figure 17:**
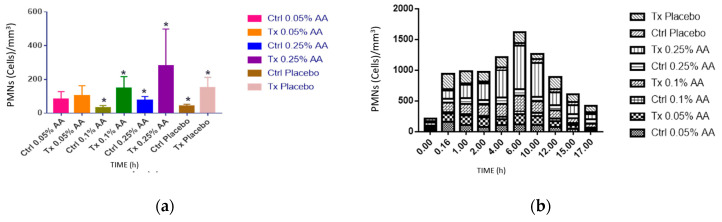
(**a**) The columns represent the different controls with their treatment. The * indicates those groups in which there is a statistically significant difference (*p* < 0.05); (**b**) the columns indicate the PMN count for each control and treatment.

The first part consisted of the selection of 4 animals for the determination of the duration of the inflammatory model and the measurement of the degree of inflammation produced by the OI from the PMN count. The average results are found in Table 14 and Figure 17 where the number of PMNs (cells/mm^3^) is observed, with a statistically significant difference (Table 15) between the AA at 0.25% and the placebo, so this concentration was chosen for the rest of the experiment.

The results in Figure 17b show the diminution of PMN cells after 6 h after OI placement. This allowed us to determine the sampling time.

**Figure 18:**
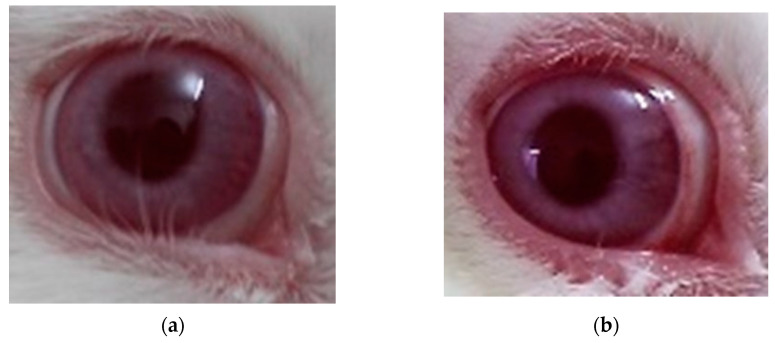

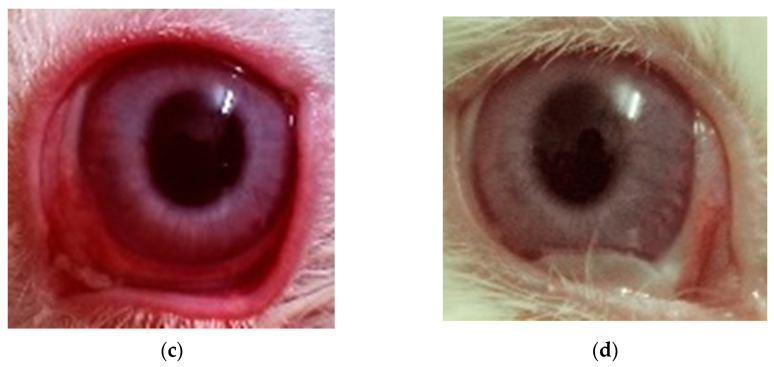
Determination of the inflammatory model. (**a**) Administration of AA at 0.05%; (**b**) AA at 0.1%; (**c**) AA at 0.25%; (**d**) OI placebo.

Figure 18 shows the irritation caused by exposure to different concentrations of AA; the highest dose is 0.25%, where changes in the eyes of rabbits can be better seen (Figure 18c)

### 3.19. Comparison of OD and OI in the Inflammation Model

After the establishment of the AA concentration to use (0.25%) and the inflammation caused by placing the insert, in addition to determining the sampling times, the rest of the experiment was performed by dividing the rabbit population into two groups randomly (8 for each group): OD of DEX was instilled in one group and the OI was administered in the other. The Table 16 results are the average of the PMNs counted in the different samples obtained.

**Figure 19:**
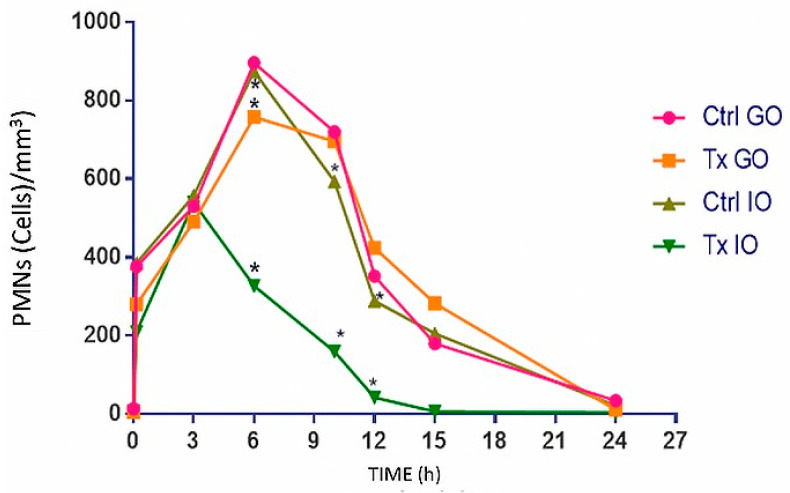
Comparative results between both pharmaceutical forms (OD–OI). It is observed that both OD and OI decrease inflammation, however, when performing the paired t test (*p* < 0.05), there is a statistically significant difference between the groups that have *.

**Figure 20:**
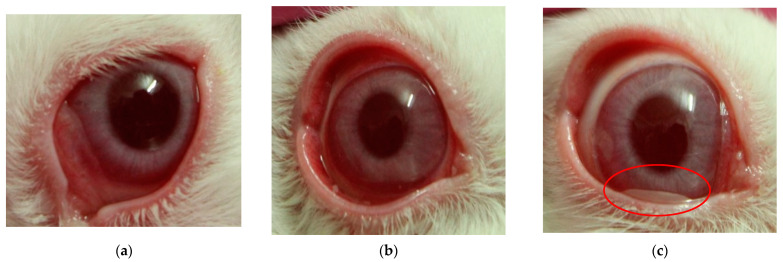
Comparative photographs of inflammation at 6 h sampling. (**a**) Control (AA 0.35%); (**b**) treatment with OD; (**c**) treatment with OI: the arrow points to the OI placed in the conjunctival sac of the rabbit.

The PMN cell count was realized in a Neubauer chamber, then the statistical analysis was performed to determine the presence or absence of the statistically significant difference between groups (Table 17). Figure 19 shows the results of the different groups and those with significant differences are marked with a *. In Figure 20 can be observed the inflammation caused by AA and the effect of both pharmaceutical forms.

## 4. Discussion

As a result, innovation in the development of ophthalmic drug delivery systems has been carried out with the aim of increasing the bioavailability of active ingredients, which is reflected in the improvement of patients. The application of a design of experiments allowed us to evaluate and determine the critical factors in the process, obtaining a formulation with optimal characteristics and with a low manufacturing cost. Compared to previous investigations with DEX-loaded eye films like Ghezzi, et. al. [30], in addition, another active compound was used as well as more excipients.

Based on the organoleptic characteristics, mixture 7 (Table 2) was chosen, as it had tactile and visual properties suitable for handling during the mechanical tests. The formulations were poured into different molding surfaces to determine which of them could be easily taken off for further manipulation and characterization.

A response surface design is a set of advanced DOE techniques that help better understand and optimize response. Response surface design methodology is frequently used to refine models after having determined important factors using screening designs or factorial designs, especially if curvature is suspected on the response surface [31]. The effects of independent variables or study variables (Xi) on dependent or response variables (Yi) were evaluated by using the statistical program Statgraphics Centurion XV.II.

With the results obtained from the design of experiments, we managed to optimize the formulation, which was physiochemically sterilized to ensure that it had the appropriate characteristics to act as a pharmaceutics form of ophthalmic administration. From this moment on, the rest of the tests were performed with the optimal formulation, starting with the determination of the drug’s ability to cross biological membranes, allowing us to know the release kinetics for both the uptake of the drug and its diffusion through the membrane used: conjunctival sac and eyeball. Franz-type cells were used in the investigation of the permeation of the formulation, so that a good correlation between the release of the drug in vitro and the permeations ex vivo can be indicative of a good correlation ex vivo and in vivo [32].

The results show that the cumulative percentages of DEX in each structure present no statistically significant difference (*p* < 0.05), so it is established that the drug is not crossing the biological membrane, limiting the effect to the application site, thus minimizing systemic effects. Likewise, the amount released by the optimal formulation (13.5% = 1.007 mg DEX) is sufficient to reach the therapeutic dose to have an anti-inflammatory effect. To confirm the results obtained during the in vitro–ex vivo tests, in vivo studies were performed to establish the correlation between these characterization tests with an animal model.

The choice of sterilization method is important as polymers can be sensitive to various techniques, the effect of sterilization being a key factor in the development of the product [33,34]. Taking into account the above considerations, the use of UV radiation was chosen as a sterilization method as it has been found that this technique does not significantly modify the properties of the products once they have already polymerized [35,36,37]. It was observed that when performing the evaluation of microbial growth in both TSA and CB culture media, in the first 0 to 10 min of radiation there is already a development of microorganisms. Observations were made over 14 days, showing that microbial growth is no longer observed after 20 min of UV radiation time, so this time was chosen to sterilize the OI.

As the last stage of the experimental strategy, in vivo tests were performed using 20 animals (New Zealand albino rabbits) with an average weight between 1.5–2 kg. For the development of the inflammatory model caused by arachidonic acid (AA), the concentration of 0.25% was established, as it is within this that statistically significant differences are found with respect to other concentrations (*p* < 0.05) [29,38]. The duration of inflammation was established by determining the time in which there was a greater migration of PMN cells, being 6 h; therefore, sampling was performed at 0 min, 10 min, and later 3, 6, 10, 12, 15, and 24 h. Finally, DEX was administered by OD and OI, showing that there is a statistically significant difference between both pharmaceutical forms (*p* < 0.05). The results show that when the DEX release begins and the therapeutic concentrations are reached, the inflammation was also reduced, and these results show that the greatest challenge within eye therapy is the maintenance of therapeutic concentrations at the target site, taking into account the anatomical and physiological constraints of said anatomical structure such as tearing, nasolacrimal drainage, low uptake in the conjunctiva, and poor corneal permeability, especially of water-soluble drugs, which allow only a fraction of the administered dose (less than 1%) to be absorbed into the eye. Requiring frequent administration of conventional pharmaceutical forms implies very high drug concentrations resulting in severe eye effects in addition to the possibility of systemic effects [31].

## 5. Conclusions

In the present study, the anti-inflammatory effect of DEX eye inserts as a new pharmaceutical form of veterinary use was compared to conventional drip therapy on the market. For this purpose, mechanical and physicochemical tests and in vitro and ex vivo studies were performed, which allowed establishing that the concentrations and interactions of the polymers used (PVPK30 and ERS100) have an impact on properties: mainly on mass variation, pH, tensile strength, and the mucoadhesion strength ex vivo. The release tests conclude that the DEX release (1.0007 mg) is necessary to achieve the therapeutic effect for at least 5 days after the placement of the ophthalmic insert. Further, permeation tests showed that the pharmaceutical form has a local effect by not allowing the active substance to reach systemic circulation. The sterilization technique used for OI was UV radiation for 20 min. Therefore, the in vivo studies in rabbits obtained better results by placing ophthalmic inserts than with the instillation of drops as the conventional therapy, because the residence time of the drops is much shorter compared to the ocular insert. This was demonstrated in the PMN cell count.

The present study confirms the potential use of polymeric systems using PVPK30 and ERS100 as a new strategy of controlled release of ophthalmic drugs for human and veterinary use, by controlling and prolonging the release of DEX at the affected site by decreasing the systemic effects of the drug. Even more, the use of easily replicable processes, the design of experiments, and low-cost materials give an advantage in comparison to those already existing in the market or those under investigation since they have more components (Table 18).

## Figures and Tables

**Table 1:** Sites that require medication [19].

Site	Tissues Involved	Drug Example
Surface structures	Eyelids Conjunctive Cornea	Antimicrobial Anti inflammatory
Iris	Muscles for contraction or dilation	Mydriatics Myotics
Ciliary body	Aqueous ephytelium	Hypotensive agents
Posterior segment	Vitreous Retina	Immunosuppressive agents
Retrobulbar fabric	Muscles, nerves, and connective tissue	An aesthetic agent

**Table 2:** Proposed polymers for the formulation of eye inserts.

Polymers	
Polyvinylpyrrolidone K-30 (PVPK30)	Eudragit RS100^®^ (ERS100)
Hypromellose	Kollicoat IR^®^
Polyvinyl alcohol (PVA)	Pluronic F-127^®^ (PF127)
Hyaluronic acid	Chitosan

**Table 3:** Proposed mixtures for film formulation.

Mixture 1					Mixture 2		
Excipient	% (w/v) 1	% (w/v) 2	% (w/v) 3	% (w/v) 4	Excipient	% (w/v) 1	% (w/v) 2
HPMC	0.45	1	0.45	1	PVA	0.25	3
Pluronic F-127^®^	5	5	10	10	HPMC	0.45	1
Sodium Benzoate	0.5	0.5	0.5	0.5	Sodium Benzoate	0.5	0.5
DEX	1.5	1.5	1.5	1.5	Pluronic F-127^®^	5	5
Water c.b.p.	100	100	100	100	DEX	1.5	1.5
					Water c.b.p.	100	100
Mixture 3					Mixture 4		
Excipient	% (w/v) 1		% (w/v) 2		Excipient	% (w/v) 1	% (w/v) 2
Pluronic F-127^®^	5		5		PVA	2	2
HPMC	1		1		HPMC	1	1
PVA	1.5		2		PVP	1	5
Hyaluronic ac.	0.3		0.3		PEG	1.6	1.6
Sodium Benzoate	0.5		0.5		Sodium Benzoate	0.5	0.5
DEX	1.5		1.5		DEX	1.5	1.5
Water c.b.p.	100		100		Water c.b.p	100	100
Mixture 5					Mixture 6		
Excipient	% (p/v) 1		% (w/v) 2		Excipient	% (w/v) 1	% (w/v) 2
Chitosan	3		3		Chitosan	3	3
Lactic ac.	1		2		Acetic ac.	1	2
DEX	1.5		1.5		DEX	1.5	1.5
Mixture 7					Mixture 8		
Excipient	% (w/v)				Excipient	% (w/v)	
Eudragit ^®^ RS100	5				Eudragit RS^®^ 100	5	
PVP K-30	10				Kollicoat^®^ IR	10	
Triethycitrate	15 *				Triethycitrate	15 *	
DEX	1.5				DEX	1.5	

* a precipitate is formed.

**Table 4:** Components of the ophthalmic films.

Component	% (w/v)	Function
DEX	1.5	Active ingredient
Eudragit RS^®^ 100	1–5	Film agent
Propyleneglycol	20	Cosolvent/Conservative
Triethycitrate	15 *	Plasticizer
Boric acid	1.404	Isotonizan/ Conservative Agent
Water–Ethanol	70/30	Dissolution medium

* a precipitate is formed.

**Table 5:** Central composite design properties (CCD).

Experimental Factors	2
Number of blocks	3
Number of responses	10
Number of runs	11, including 3 central points per block
Degrees of freedom for error	5

**Table 6:** Factors and responses measured in CCD.

Factors	Variables under Study		Responses
Eudragit ^®^ RS100	Low	1%	Weight (mg) Thickness (mm) pH Tensile strength (g) Humidity (%) Mucoadhesion strength ex vivo (g) Ex vivo mucoadhesion time (h) Chemical Content (%) In vitro release (%) Swelling (%)
	High	5%	
PVP K-30	Low	3.5%	

**Table 7:** Physicochemical characterization of ocular inserts.

Formulation	Weight Variance (mg)	Thickness Variance (mm)	pH	Tensile Strength (gF)	Hu (%)
F1	0.45 ± 0.08	0.44 ± 0.08	6.96 ± 0.04	11.25 ± 17.94	16.67 ± 11.07
F2	0.35 ± 0.08	0.28 ± 0.08	7.04 ± 0.04	18.25 ± 17.94	21.46 ± 11.07
F3	0.33 ± 0.08	0.43 ± 0.08	7.03 ± 0.04	15.50 ± 17.94	25.32 ± 11.07
F4	0.20 ± 0.08	0.24 ± 0.08	7.00 ± 0.04	71.16 ± 17.94	42.28 ± 11.07
F5	0.27 ± 0.08	0.27 ± 0.08	7.11 ± 0.04	20.50 ± 17.94	37.80 ± 11.07
F6	0.29 ± 0.08	0.40 ± 0.08	6.96 ± 0.04	32.83 ± 17.94	24.73 ± 11.07
F7	0.38 ± 0.08	0.25 ± 0.08	6.99 ± 0.04	24.50 ± 17.94	17.57 ± 11.07
F8	0.37 ± 0.08	0.29 ± 0.08	7.01 ± 0.04	12.50 ± 17.94	16.30 ± 11.07
F9	0.21 ± 0.08	0.20 ± 0.08	7.01 ± 0.04	7.50 ± 17.94	37.77 ± 11.07
F10	0.42 ± 0.08	0.33 ± 0.08	6.99 ± 0.04	36.00 ± 17.94	43.33 ± 11.07
F11	0.36 ± 0.08	0.24 ± 0.08	7.01 ± 0.04	14.66 ± 17.94	14.99 ± 11.07

**Table 8:** In vitro evaluation of ocular inserts.

Formulation	Mucoashesion Strength (gF)	Mucoadhesion Time * (h)	Chemical Content (%)	Release Drug (%)	Swelling (g)
F1	9 ± 3.82	120	95.71 ± 1.10	11.55 ± 1.42	0.07 ± 0.03
F2	16 ± 3.82	120	98.00 ± 1.10	14.20 ± 1.42	0.08 ± 0.03
F3	6.5 ± 3.82	120	97.24 ± 1.10	9.80 ± 1.42	0.05 ± 0.03
F4	8 ± 3.82	120	98.77 ± 1.10	13.11 ± 1.42	0.14 ± 0.03
F5	17.5 ± 3.82	120	98.58 ± 1.10	11.77 ± 1.42	0.05 ± 0.03
F6	12.5 ± 3.82	120	95.71 ± 1.10	11.66 ± 1.42	0.06 ± 0.03
F7	10.5 ± 3.82	120	98.39 ± 1.10	13.95 ± 1.42	0.09 ± 0.03
F8	8.5 ± 3.82	120	98.39 ± 1.10	14.50 ± 1.42	0.1 ± 0.03
F9	6.5 ± 3.82	120	97.24 ± 1.10	11.83 ± 1.42	0.06 ± 0.03
F10	13.5 ± 3.82	120	96.86 ± 1.10	11.95 ± 1.42	0.03 ± 0.03
F11	7.5 ± 3.82	120	98.20 ± 1.10	13.24 ± 1.42	0.07 ± 0.03

* The test was performed for 120 h without observing any mucosal detachment, so no standard deviation value was reported.

**Table 9:** Ex vivo MS values.

Formulation	Conjuctival Sac (SC) (gF)	Eyeball (GOC) (gF)
F1	9	4
F2	16	4.5
F3	6.5	6
F4	8	5
F5	17.5	8.5
F6	12.5	5
F7	10.5	4.5
F8	8.5	5.5
F9	6.5	6
F10	13.5	8.5
F11	7.5	4

**Table 10:** Optimization of the formulation.

Factor	Low	High	Optimus
Eudragit^®^ RS100	0.17157	5.82843	0.171517
PVP K-30	2.15381	11.3462	6.4554
**Expected responses**			
ThV		0.214274 mm	
MS		13.2152 gF	
DS		12.6596%	
Sw		0.0558292 g	

**Table 11:** Results of the optimization phase of ophthalmic films.

Factors	Response
Thickness (mm)	0.265 ± 0.095
pH	7.11 ± 0.040
Tensile strength (gF)	15.50 ± 3.940
% Humidity	22.54 ± 1.700
Mucoadhesion Strength (gF)	16.89 ± 3.460
Chemical content (%)	98.19 ± 1.124
Drug Release (%)	13.51 ± 0.710
Sw (g)	0.0403 ± 0.023

**Table 12:** Accumulated quantity (mg) and percentage (%) of DEX in the conjunctival sac and eyeball for 48 h using Franz-type cells.

Time (h)	Conjunctival Sac	Eyeball	Conjunctival Sac	Eyeball
	Accumulated Quantity (mg)	Accumulated Quantity (mg)	Percentage (%)	Percentage (%)
1	0	0	0	0
2	0.00142	0.00285	0.01913	0.03826
4	0.00285	0.00713	0.03826	0.09567
6	0.00428	0.00999	0.05740	0.13394
8	0.00570	0.01427	0.07653	0.19134
10	0.00713	0.01570	0.09567	0.21047
12	0.00856	0.01855	0.11480	0.24874
24	0.01141	0.02283	0.15307	0.30615
28	0.01427	0.02426	0.19134	0.32528
32	0.01712	0.02569	0.22961	0.34441
48	0.01998	0.02712	0.26788	0.36355

**Table 13:** Sterility test results to determine UV radiation time.

Time of Incubation	Radiation Time (min)	Microbial Growth (MiG)	Microbial Growth TSA
DAY 1	0	without development	without development
	10	without development	without development
	20	without development	without development
	30	without development	without development
	60	without development	without development
DAY 2	0	Developing	without development
	10	Developing	without development
	20	without development	without development
	30	without development	without development
	60	without development	without development
DAY 3	0	Developing	without development
	10	Developing	without development
	20	without development	without development
	30	without development	without development
	60	without development	without development
DAY 4	0	Developing	Developing
	10	Developing	Developing
	20	without development	without development
	30	without development	without development
	60	without development	without development
DAY 5	0	Developing	Developing
	10	Developing	Developing
	20	without development	without development
	30	without development	without development
	60	without development	without development
DAY 10	0	Developing	Developing
	10	Developing	Developing
	20	without development	without development
	30	without development	without development
	60	without development	without development
DAY 14	0	Developing	Developing
	10	Developing	Developing
	20	without development	without development
	30	without development	without development
	60	without development	without development

**Table 14:** Measurement of AA-induced inflammation: 0.05%, 0.1%, 0.25%, and placebo OI.

Time (h)	0.05% Ac. Aq.		0.1% AA		0.25% Ac. Aq		Placebo	
	PMNs (cells/mm^3^)		PMNs (cells/mm^3^)		PMNs (cells/mm^3^)		PMNs (cells/mm^3^)	
	Ctrl	Tx	Ctrl	Tx	Ctrl	Tx	Ctrl	Tx
0	21.25	12.5	18.5	22.1	25	43.75	30	40
0.16	155	142.5	20.3	154.3	58.75	133.75	26.25	252.5
1	107.5	158.75	19.7	160.89	96.25	213.75	30	198.75
2	76.25	155	26.4	180.16	80	267.5	27.5	160
4	103.75	100	34.63	212.2	105	441.25	52.5	162.5
6	121.25	165	45.17	256	98.75	711.25	45	180
10	108.75	143.75	53.2	189.4	72.5	555	57.5	81.25
12	77.5	88.75	48.5	132.56	86.25	203.25	55	195
15	45.83	56.93	32.7	80.23	67.4	150.65	48.6	120.3
17	20.23	11.4	28.73	67.9	70.32	80.54	32.4	108.3

**Table 15:** Analysis of variance, ANOVA.

Source	Sum of Squares	DF	Medium Square	*p*-Value
Between groups	443,336.	7	63,333.7	0.0000
Intra groups	570,087.	72	7917.88	
Total	1.01342 × 10^6^	79		

**Table 16:** Comparative results of both pharmaceutical forms (OD, OI) with their respective control.

Time (h)	Ophthalmic Drops		Ophthalmic Inserts	
	PMNs (cells/mm^3^)		PMNs (cells/mm^3^)	
	Ctrl	Tx	Ctrl	Tx
**0**	13.750	5.625	6.250	7.500
**0.16**	376.230	280	385	209.375
**3**	530	490.125	556.875	539.375
**6**	895.625	757.500	874.375	326.875
**10**	719.375	695.625	593.750	159.375
**12**	351.875	424.375	288.125	41.875
**15**	180	282.500	205.625	6.250
**24**	34.375	11.125	21.250	3.750

**Table 17:** Analysis of variance, ANOVA.

Source	Sum of Square	DF	Medium Square	*p*-Value
Between groups	272,744.	3	90,914.6	0.3306
Intra groups	2.13496 × 10^6^	28	76,248.6	
Total	2.4077 × 10^6^	31		

**Table 18:** Differences between DEX OI and previous investigations.

OI DEX Development and Characterization	Previous Technologies
A DoE was performed, optimizing both the process and the pharmaceutical form.	No DoE was performed, so the process and pharmaceutical form are not optimized.
Only two polymers and DEX are used for the development of eye inserts.	In addition to DEX, they use hyaluronic acid and levofloxacin.
The characterization process is more extensive when carrying out tests of tensile strength, bioadhesion, bioadhesion postwetting, chemical content, release, etc.	Tests on ophthalmic films only included swelling tests.
Other technologies such as nanoparticles were not used to contain or modify DEX release.	They make use of nanometric technology for DEX application and management.

## Data Availability

The original contributions presented in the study are included in the article, further inquiries can be directed to the corresponding author/s.

## Abbreviations

AA	Arachidonic acid
OI	Ophthalmic insert
OD	Ophthalmic drops
DEX	Dexamethasone
TSA	Soybean trypticase agar
NDS	Nasolacrimal system
PMN	Polymorphonuclears cells
AP	Active principle
DOE	Design of experiments
PLF	Precorneal lacrimal film
PVPK30	Polyvinyl pyrrolidone K30
ERS100	Eudragit RS100
PVA	Polyvinyl alcohol
PF127	Pluronic F 127
CCD	Central composite design
Wv	Weight variation
Th	Thickness
Ts	Tensile strength
Hu	Humidity
MS	Mucoadhesion strength
MT	Mucoadhesion Time
Sw	Swelling
ChC	Chemical Content
ThV	Thickness variation
